# Dissecting Detergent-Insoluble Proteome in Alzheimer's Disease by TMTc-Corrected Quantitative Mass Spectrometry

**DOI:** 10.1016/j.mcpro.2023.100608

**Published:** 2023-06-24

**Authors:** Masihuz Zaman, Yingxue Fu, Ping-Chung Chen, Huan Sun, Shu Yang, Zhiping Wu, Zhen Wang, Suresh Poudel, Geidy E. Serrano, Thomas G. Beach, Ling Li, Xusheng Wang, Junmin Peng

**Affiliations:** 1 Department of Structural Biology, St Jude Children's Research Hospital, Memphis, Tennessee, USA; 2 Department of Developmental Neurobiology, St Jude Children's Research Hospital, Memphis, Tennessee, USA; 3 Center for Proteomics and Metabolomics, St Jude Children's Research Hospital, Memphis, Tennessee, USA; 4 Banner Sun Health Research Institute, Sun City, Arizona, USA; 5 Department of Biology, University of North Dakota, Grand Forks, North Dakota, USA

**Keywords:** Alzheimer's disease, neurodegenerative disease, insoluble proteome, amyloidome, splicing, proteomics, proteome, mass spectrometry, tandem mass tag, TMT complement ions

## Abstract

Protein aggregation of amyloid-β peptides and tau are pathological hallmarks of Alzheimer's disease (AD), which are often resistant to detergent extraction and thus enriched in the insoluble proteome. However, additional proteins that coaccumulate in the detergent-insoluble AD brain proteome remain understudied. Here, we comprehensively characterized key proteins and pathways in the detergent-insoluble proteome from human AD brain samples using differential extraction, tandem mass tag (TMT) labeling, and two-dimensional LC–tandem mass spectrometry. To improve quantification accuracy of the TMT method, we developed a complement TMT–based strategy to correct for ratio compression. Through the meta-analysis of two independent detergent-insoluble AD proteome datasets (8914 and 8917 proteins), we identified 190 differentially expressed proteins in AD compared with control brains, highlighting the pathways of amyloid cascade, RNA splicing, endocytosis/exocytosis, protein degradation, and synaptic activity. To differentiate the truly detergent-insoluble proteins from copurified background during protein extraction, we analyzed the fold of enrichment for each protein by comparing the detergent-insoluble proteome with the whole proteome from the same AD samples. Among the 190 differentially expressed proteins, 84 (51%) proteins of the upregulated proteins (n = 165) were enriched in the insoluble proteome, whereas all downregulated proteins (n = 25) were not enriched, indicating that they were copurified components. The vast majority of these enriched 84 proteins harbor low-complexity regions in their sequences, including amyloid-β, Tau, TARDBP/TAR DNA-binding protein 43, SNRNP70/U1-70K, MDK, PTN, NTN1, NTN3, and SMOC1. Moreover, many of the enriched proteins in AD were validated in the detergent-insoluble proteome by five steps of differential extraction, proteomic analysis, or immunoblotting. Our study reveals a resource list of proteins and pathways that are exclusively present in the detergent-insoluble proteome, providing novel molecular insights to the formation of protein pathology in AD.

## Highlights

• Development of a TMTc-based strategy to correct for ratio compression.
• Meta-analysis of detergent-insoluble proteome datasets.
• Enrichment analysis in detergent-insoluble fraction compared with brain lysate.
• Identification of 84 enriched proteins accumulated in AD.
• The vast majority of the 84 proteins containing low-complexity regions.

## Graphical Abstract

**Figure:**
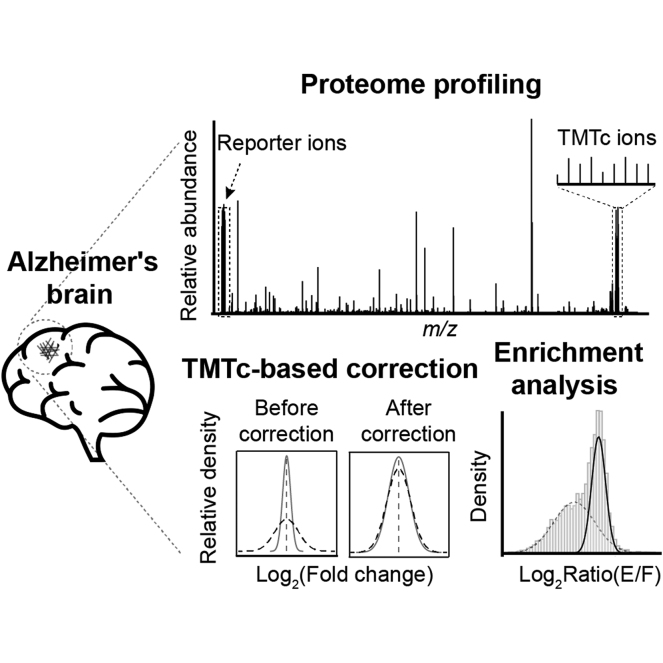

## In Brief

Using the TMT-LC/LC–MS/MS method and a TMTc-based strategy to correct for ratio compression, we analyzed detergent-insoluble proteome from 30 human AD and control brain samples. We identified 84 proteins enriched in the detergent-insoluble fraction, accumulated in AD, and mostly containing low-complexity regions. Our study highlights AD pathological hallmarks resistant to detergent extraction, providing a list of proteins and pathways exclusively present in the detergent-insoluble proteome.

Alzheimer's disease (AD) is a progressive and currently incurable neurodegenerative disease (1, 2, 3) that affects more than 58 million individuals worldwide (4). The most common clinical manifestations of AD include irreversible memory loss, progressive cognitive decline, and impaired reasoning accompanied by loss of functional autonomy (5). Two neuropathological hallmarks of AD are the presence of senile plaques and neurofibrillary tangles comprised of detergent-insoluble amyloid-beta (Aβ) and phosphorylated-tau, respectively (6, 7). Other proteins associated with AD senile plaques and neurofibrillary tangles were explored by laser capture microdissection and mass spectrometry (MS) (8, 9, 10, 11). The Aβ accumulation remains a major target for the development of disease-modifying therapies (3, 12). Therefore, the analysis of protein components in detergent-insoluble proteome from AD patients could elucidate pathogenic mechanisms underlying AD, permitting possible strategies for disease prevention and treatment.

While numerous efforts have been made to analyze the whole proteome and the identification of key proteins and pathways implicated in AD pathogenesis (13, 14, 15, 16, 17, 18, 19), only a few studies have focused on proteins in detergent-insoluble brain proteome from AD patients, with limited proteome coverage (20, 21, 22, 23, 24). The detergent-insoluble proteome in AD was initially analyzed using sequential fractionation combined with gel electrophoresis and LC–MS/MS, leading to the identification of 512 proteins, with 11 found to be increased in AD samples (20). Another more in-depth label-free analysis of the brain insoluble proteome was performed, identifying 4216 proteins with 36 differentially expressed proteins (DEPs) in AD, including Aβ, tau, APOE, complement components, and some novel components of RNA splicing dysfunction, such as U1 small nuclear ribonucleoprotein (snRNP) (21). The U1 snRNP aggregation and pathology were validated by several subsequent biochemical and histochemical studies of human specimens (22, 25, 26, 27). We further generated a mouse model to support a causative role of U1 snRNP dysfunction in neurodegeneration (28).

Although TAR DNA-binding protein 43 (TDP-43) aggregation was originally discovered in the brains of patients with frontotemporal lobar degeneration and amyotrophic lateral sclerosis (29), the TDP-43 pathology was identified in approximately 20 to 50% of individuals with AD (30), and this number increased to 75% when only high-stage AD cases were included (31), leading to the proposal of a disease entity known as limbic-predominant age-related TDP-43 encephalopathy (32). In addition to U1 snRNP and TDP-43, numerous other proteins aggregated in the detergent-insoluble proteome remain to be characterized.

The quantitative proteomics strategy employing tandem mass tag (TMT) chemical labeling coupled with two-dimensional LC–tandem MS (TMT-LC/LC–MS/MS) enables the detection of over 10,000 unique proteins (19, 33). The enhancement in protein identification can be largely attributed to (i) the advancement of the latest MS instruments with rapid scanning rates (34), (ii) efficient two-dimensional LC fractionation, achieved by combining high pH and low pH reverse-phase LC with sub-2 μm resin (35, 36), and (iii) enhanced database search engines including MSFragger/FragPipe (37, 38), real-time searching (39), and other hybrid methods, such as Byonic (40), JUMP method (41), and PEAKS DB (42), which combine *de novo* amino acid tag identification and pattern-matching functions.

Compared with the label-free proteomics, the TMT-LC/LC–MS/MS strategy reduces inter-run variability and enhances multiplexing capacity, which substantially improve the throughput of quantitative proteomics (43). For instance, we developed a 27/29-plex TMT-LC/LC–MS/MS assay to analyze human AD samples (44, 45). However, the TMT strategy has a limitation of ratio compression because of peptide coelution during the LC–MS quantification (46). To address this limitation, several methods have been proposed, such as extensive LC fractionation (36), MS3 method (46), gas phase purification (47), and computational correction (36, 48). However, the additional steps in the MS3 and gas phase purification methods decrease data acquisition speed and detection sensitivity, resulting in a reduced number of quantified proteins (46, 47). More recently, a method based on complement TMT (TMTc) reporter ions in MS2 scans has emerged as a promising approach to improve the accuracy of TMT-based quantification, although not every peptide can generate sufficient TMTc ions (49, 50, 51).

In this study, we aim to comprehensively characterize key proteins and pathways implicated in AD by profiling detergent-insoluble brain proteome from AD patients. To achieve this goal, we first improved our profiling strategy combining sequential centrifugation and TMT-LC/LC–MS/MS platform. We then developed an integrated TMT strategy, using both reporter ion– and TMTc ion–based quantification to improve the quantitative accuracy of the resulting detergent-insoluble proteome. Finally, we validated altered proteins in the detergent-insoluble proteome by confirming their enrichment during differential protein extraction.

## Experimental Procedures

### Experimental Design and Statistical Rationale

Experiments were performed using cell lysates from *Escherichia coli*, mouse brain, and human postmortem brain samples. The MS proteomics data were deposited to the ProteomeXchange Consortium *via* the PRIDE (52) partner repository with the dataset identifier PXD038381 and to the MassIVE proteomics data repository (53) with the dataset identifier MSV000091796. To evaluate reproducibility and quantitative accuracy, experimental replicates were conducted and subjected to statistical analysis using the reported methods. The experimental design and statistical rationale can be found in the corresponding figure legends and main text, which include the application of Fisher's exact test (*p* value) for pathway enrichment and false discovery rate (FDR) analysis utilizing the Benjamini–Hochberg procedure.

### Human Postmortem Brain Tissue

Deidentified human postmortem brain tissue samples (frontal gyrus) were provided by the Brain and Body Donation Program at Banner Sun Health Research Institute. The program conducts annual standardized clinical assessments and has obtained approval from the Institutional Review Board, including informed consent and protocol. Clinical and pathological diagnoses were established based on the relevant criteria (54).

### Protein Differential Extraction, Quantification, and Digestion

Based on a previously reported protocol (21), human brain samples (100 mg each, five controls and five AD cases) were homogenized in low salt buffer (1:10 w/v ratio, 50 mM Hepes, pH 7.5, 0.1 M NaCl, 1 mM EGTA, 10% sucrose, 1× Protease inhibitor) with glass beads (∼200 μl) using Bullet blender (24 Gold, 30 s × seven cycles). The homogenates were transferred in another Eppendorf tube to adjust NaCl to 0.8 M and sarkosyl (*N*-lauroyl-sarcosine) to 1% (w/v), followed by sonication for 20 s (power 40%, 1 s on, 1 s off) on ice using a probe sonicator. Aliquots of the sonicated lysates were taken as input, whereas the remaining were centrifuged at 5000*g* for 5 min (pellet 1), 10,000*g* for 5 min (pellet 2), 20,000*g* for 10 min (pellet 3), and 130,000*g* for 30 min at 4 °C (pellet 4). Finally, we resuspended pellet 4 in extraction buffer (50 mM Hepes, pH 7.5, 10% sucrose, 0.1 M NaCl, and 0.2% sarkosyl), save 25% as an aliquot (pellet 4), and performed another ultracentrifugation at 130,000*g* for 30 min at 4 °C (pellet 5). The insoluble pellets were resuspended in 8 M urea containing 0.5% sodium deoxycholate and 50 mM Hepes, pH 7.5, for Western blotting and proteome profiling. All experiments were carried out in triplicates.

For silver staining the SDS gel, total homogenate and pellet fractions were resolved on SDS-PAGE (4–20%). The gel was fixed for 10 min with 50% (v/v) methanol and 7% (v/v) acetate acid and then washed twice (5 min each) with water and rinsed in water at 4 °C overnight. Next, we soaked the gel with 0.02% Na_2_S_2_O_3_ gel for 1 min, washed twice for 30 s, stained for 10 min with 0.1% AgNO_3_, and developed with fresh 0.05% formaldehyde (v/v) and 3% Na_2_CO_3_ for about 10 min until the bands were visible. The reaction was quenched with 5% acetic acid (v/v). The concentration of each sample was estimated by silver-stained short gel (<1 cm) with bovine serum albumin as a standard (55).

For TMT analysis, the quantified protein samples (∼20 μg per sample) were resolved on a short SDS gel (10% w/v, <1 cm) and stained with Coomassie blue. Each gel lane was excised into small 1 mm^3^ pieces, and Cys residues were reduced and alkylated by iodoacetamide (10 mM), followed by a modified digestion protocol with trypsin (1:50 [w/w]) overnight at 37 °C (56, 57). To be compatible with the following TMT labeling, we used 10 mM Hepes buffer (pH 8.5) to replace ammonium bicarbonate during regular in-gel digestion.

### TMT Labeling, Pooling, and Fractionation of Human Brain Samples

The experiments were performed using a published protocol (44) with slight modifications. In-gel digested peptide samples were resuspended into 50 mM Hepes buffer (pH 8.5) and labeled with 18-plex TMTpro reagents (TMT/peptide ratio of 2.5:1) for 30 min at 21 °C. The TMT labeling efficiency was assessed for each sample by desalting ∼1 μg of both TMT-labeled and prelabeled samples and analyzing them under the same LC–MS/MS conditions. Next, the top 10 peptide ion peaks in the MS1 spectrum of the prelabeled samples were manually identified and confirmed to be undetectable in the TMT-labeled samples. After confirming complete labeling, the samples were quenched with 5% hydroxylamine for 15 min at 21 °C, equally pooled, and desalted. The pooled TMT labeled peptides were fractionated by an offline basic pH reverse-phase LC (buffer A: 10 mM ammonium formate, pH 8.0, in water, buffer B: buffer A in 90% acetonitrile). Two LC columns were used: a Waters XBridge C18 column (3.5 μm beads, 1.0 mm × 50 mm) and a Waters Acquity BEH C18 column (1.7 μm beads, 2.1 mm × 150 mm). We collected a total of 320 fractions by utilizing high pH reversed-phase LC. These 320 fractions were combined into 40 superfractions by merging early, middle, and late LC fractions. For example, superfraction 1 incorporated fractions 1, 41, 81, 121, 161, 201, 241, and 281; superfraction 2 combined fractions 2, 42, 82, 122, 162, 202, 242, and 282, and so forth. The application of concatenated superfractions has been demonstrated to enhance protein sequence coverage and streamline sample processing (58, 59).

### Analysis of Fractionated Sample by Acidic LC–MS/MS

The fractionated samples were processed by acidic reverse-phase LC–MS/MS coupled with a Q Exactive HF Orbitrap MS (Thermo Fisher Scientific), using a self-packed column (75 μm × 150 mm, 1.9 μm C18 resin from Dr Maisch GmbH, at 65 °C). Peptides were eluted with 70 min gradient (buffer A: 0.2% formic acid, 5% dimethyl sulfoxide; buffer B: buffer A plus 65% acetonitrile). The setting of MS involved positive ion mode and data-dependent acquisition (top20: one MS1 scan followed by 20 MS/MS scans). MS1 scans were executed at a 60,000 resolution, 460 to 1600 *m*/*z* scan range, a maximum ion time of 50 ms, and 1 × 10^6^ automatic gain control. MS2 scans were acquired at a 60,000 resolution, a fixed initial mass of 120 *m*/*z*, a maximum ion time of 120 ms, and 1 × 10^5^ automatic gain control. The fragmentation settings included 1.0 *m*/*z* isolation window with 0.2 *m*/*z* offset, normalized collision energy of 32, and 15 s of dynamic exclusion.

### Protein Identification and Quantification by JUMP Software

The identification and quantification of peptides/proteins were accomplished by the JUMP software (36, 41). The protein database was compiled by merging protein sequences from TrEMBL, UCSC, and Swiss-Prot databases (human: 83,955 entries; mouse: 59,423 entries; downloaded in April 2020). This curated target database was then concatenated with a decoy database, whereas the target protein sequences were reversed to create a decoy database for assessing FDR (60). The mass tolerance for precursor ions and fragment ions was set at 15 ppm and 20 ppm, respectively, for database search. A maximum of two miscleavage sites were allowed for each peptide. TMTpro labeling (Lys or N terminus) and Cys carbamidomethylation were designated as static modifications, whereas Met oxidation was set as a dynamic modification. Protein FDR was kept below 1% by applying filters based on mass accuracy and JUMP-based matching scores (Jscore and ΔJn). Based on the rule of parsimony, a shared peptide by multiple proteins was typically assigned to the canonical protein form. If no canonical form is defined in the Swiss-Prot database, the peptide was assigned to the protein with the highest peptide-spectrum match (PSM) number. Annotated spectra were provided in the Supplemental Data section to support protein identification by a single unique peptide.

The identified PSMs/peptides/proteins were quantified by reporter ions (36) and TMTc ions (51) independently, and the reporter-based quantification was corrected for ratio compression according to the TMTc-based quantification. The process of TMTc-based quantification was highlighted here: (i) for each PSM, infer the theoretical and relative abundance of overlapped TMTc peaks by considering peptide isotopic envelope, MS1 isolation window, and TMT tag impurities; (ii) extract experimental TMTc ions with at least nine monoisotopic peaks from PSM-matched MS2 scans, whereas the remaining MS2 scans (∼50% in our datasets) do not have sufficient TMTc ion peaks; (iii) compute the relative abundance of each TMT channel; and (iv) summarize PSM data into peptide/protein data. This method was originally applied to a maximum of eight channels that have distinguishable TMTc peaks (49). We further developed the method to quantify all 18 TMTpro channels, in which the quantities of some channels were merged into one value (*e.g.*, 127N, 128N, and 128C), resulting in nine TMTc-based channels in the TMTpro method.

### Pathway Enrichment by Kyoto Encylopedia of Genes and Genomes and Gene Ontology Database

The analysis of pathway enrichment to infer functional groups of proteins (enriched in given dataset) was carried out with the JUMPn software (61). The analysis was accomplished with Fisher’s exact test (*p* value) against the Kyoto Encyclopedia of Genes and Genomes pathway database, cellular component annotations, molecular functions, and Gene Ontology biological process separately. The resulting *p* values were further attuned into FDR using the Benjamini–Hochberg procedure. Those enriched pathways (FDR <0.05) are considered statistically significant.

### Protein–Protein Interaction Network Analysis

The protein interaction network analysis was performed with our reported protocol (62) with slight modifications. DEPs were superimposed onto a composite protein–protein interaction (PPI) database by combining InWeb_IM, STRING (v11), and BioPlex3 (63, 64, 65), comprising 20,485 proteins and 1,152,607 PPI connections. PPI modules were defined by three-step procedures: (i) if two proteins are from the DEP list; extract a subnetwork by retaining PPI between those two proteins, (ii) for the resulting PPI subnetwork, calculate a topologically overlapping matrix between each pair of proteins, and (iii) using the hybrid dynamic tree cutting method, modularize such network into individual modules based on the topologically overlapping matrix clustering. The name of PPI modules was assigned from the biological functions of individual proteins inside the PPI modules.

## Results

### Development of an Accurate Quantification Strategy Using TMTc-Based Correction

**Fig. 1:**
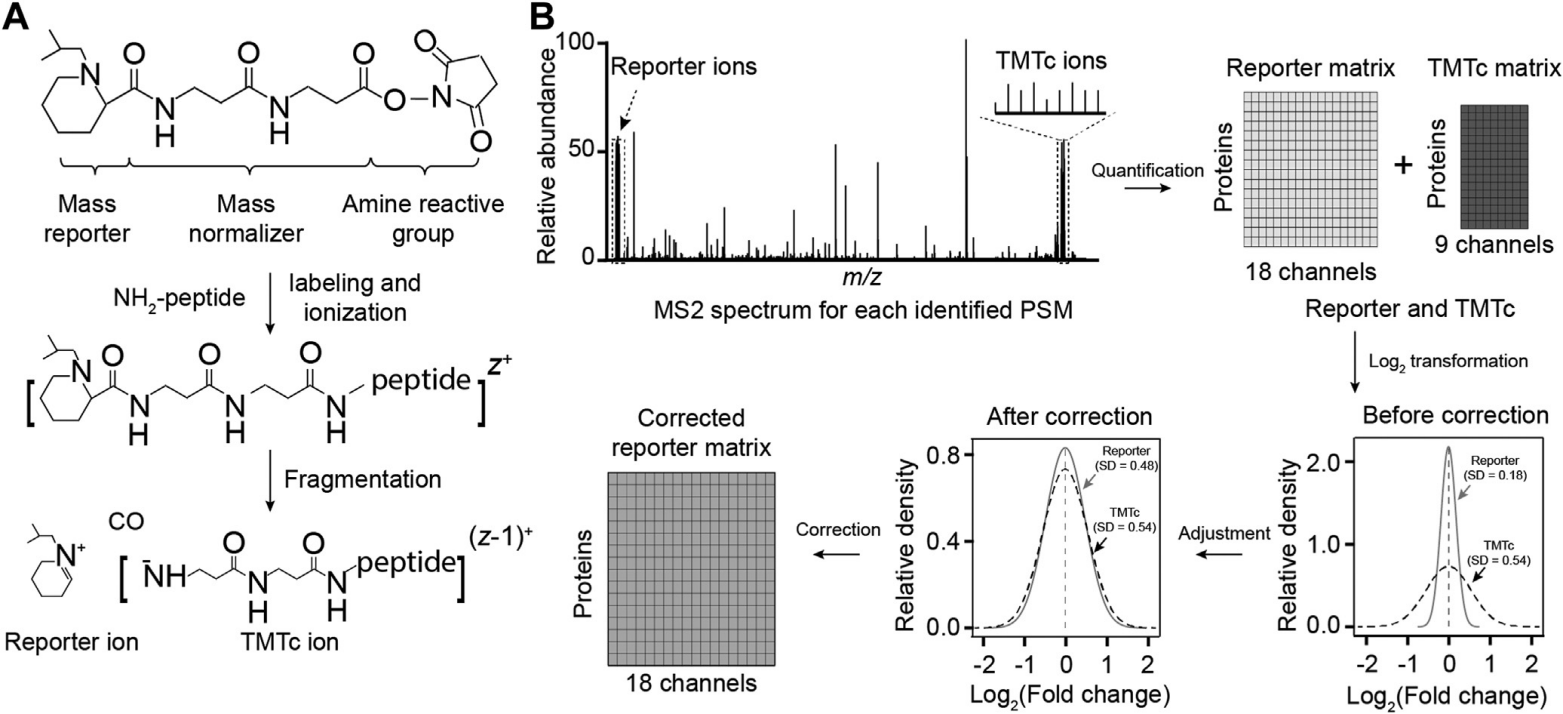
**Schematic diagram of TMTc-based correction strategy.***A*, structure of the set of 18-plex TMTpro reagents. Each reagent consists of a reporter region, a balancer region, and an amine-reactive group. During fragmentation, TMT-labeled peptides generate reporter ions, and frequently TMTc ions with neutral CO loss. *B*, TMTc-based correction strategy. TMT-labeled peptides can be quantified by both MS2 reporter ions and TMTc ions. In the quantified data matrices, the *x*-axis represents different channels, and the *y*-axis represents different proteins. The data are log transformed to derive average SD of TMT reporter– and TMTc-based matrices. Before the correction, the two SD values are vastly different because of ratio compression of report-based quantification. After correction, the two SD values become highly similar. The detailed correction steps are described in supplemental Figs. S1–S3. TMTc, complement TMT; TMT, tandem mass tag.

To accurately quantify TMT-based protein expression levels from MS2 spectra, we developed a correction strategy based on the measurement of TMTc ions. In contrast to TMT reporter ions generated from both target and coeluted ions, TMTc ions in the MS2 spectra are precursor specific, which are not impacted by coeluted and nonisobaric peptides (Fig. 1*A*). However, some TMTc ions from different TMT tags are overlapped, leading to nine TMTc ion channels from an 18-plex TMTpro set (Fig. 1*B*; supplemental Fig. S1). We and others have observed that ratio compression reduces both fold change (log_2_FC) and the experimental variations (*e.g.*, SD of log_2_FC but does not significantly impact the z values (log_2_FC/SD) (36, 66). Based on this assumption, a new strategy is proposed to correct TMT reporter ion–based protein quantification using TMTc quantitative information (Fig. 1*B*; supplemental Fig. S2).

In detail, our methodology involves calculating an SD correction factor based on the ratio of the average SD values of protein measurements (log_2_FC) from the reporter ion dataset and the TMTc ion dataset, followed by adjusting the reporter ion SD and the corresponding log_2_FC. The correction strategy encompasses four main steps. (i) Intensities of each PSM are extracted for both TMT reporter and TMTc ions, respectively, and log_2_ transformed; (ii) In both data matrices, log_2_FC for each protein and global SD for each channel are calculated by compared with the average intensities of all channels. For instance, the 18-plex TMTpro reporter ion quantification can result in 18 SD values by comparing each channel to the average of all channels; these values are then used to calculate one average SD value. In a similar manner, the TMTc ion quantification can yield nine SD values, which are also used to compute one average SD value. (iii) The SD correction factor is determined by calculating the ratio between the two average SD values. (*iv*) TMT reporter–based quantitative values of all proteins are readjusted by the SD correction factor. These steps are outlined and processed by an open-source software program that is accessible on the GitHub repository (supplemental Fig. S3).

**Fig. 2:**
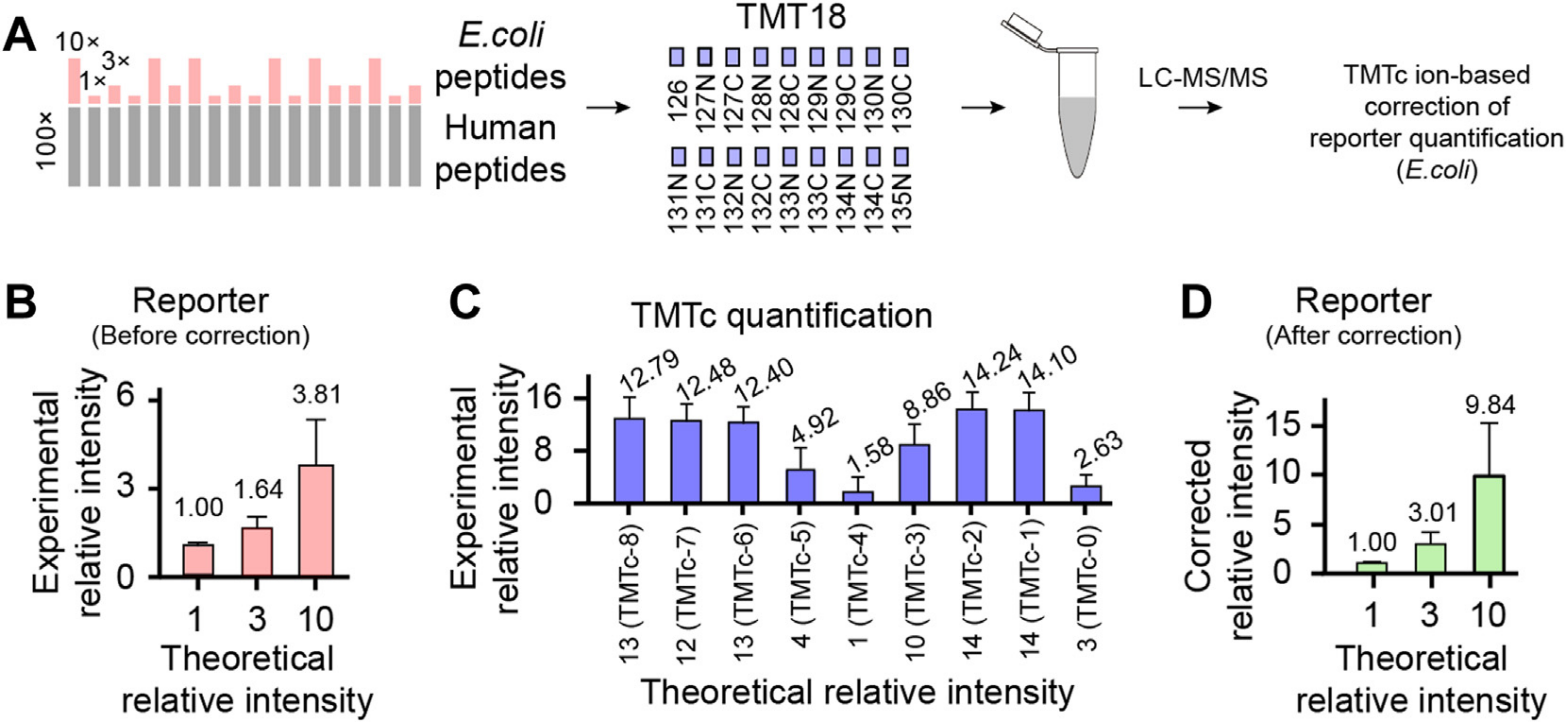
**Experimental strategy to evaluate and correct the 18-plex TMTpro data.***A*, schematic diagram of 18-plex TMTpro (TMT18) samples used in TMTc-based correction of reporter quantification. *Escherichia coli* peptides (1×, 3×, and 10×) were labeled and mixed with 100× of human peptides. The assignment of different amounts to the channels was largely random. However, the amounts of “1×” and “10×” were assigned to adjacent channels to minimize TMT channel crosstalk, since the impurity of TMT reagents often affects alternating channels. The 18 samples were then pooled together and analyzed by LC–MS/MS. *B*, experimental relative intensities quantified by TMT reporter ions in *E. coli*. *C*, experimental relative intensities quantified by TMTc ions in *E. coli*. The 18 TMTpro reagents lead to the quantification of nine TMTc channels because of some isobaric TMTc ions. *D*, experimental relative intensities of TMT reporter ions after TMTc-based correction. The error bars indicate the SDs of the analysis. TMTc, complement TMT; TMT, tandem mass tag.

To evaluate the performance of the TMTc-based correction strategy, we applied it to a TMT proteome of mixed *E. coli* and human brain lysate samples (Fig. 2*A*) as described in a previous study (36). *E. coli* peptides were labeled with the 18-plex TMTpro reagents and mixed in designated 1×:3×:10× ratios, whereas TMT-labeled human peptides were added with equal 100× in all channels as background. The mixed samples were analyzed by LC–MS/MS for quantification. TMT reporter ion–based quantification of *E. coli* peptides showed an average ratio of 1:1.64:3.81, away from the expected 1:3:10 ratios (Fig. 2*B*), whereas the TMTc-based quantification displayed expected ratios when the overlapped TMTc channels were taken into consideration (Fig. 2*C*). With the TMTc-based correction strategy, we achieved the average *E. coli* peptide ratios to 1:3.01:9.84 (Fig. 2*D*), which is close to the expected ratios of 1:3:10. This result indicates that the TMTc-based correction strategy can address the issue of ratio compression in the TMT experiments, providing an accurate estimate of protein measurement.

### Profiling Detergent-Insoluble and Whole Proteome from Human AD Brain Tissues

**Fig. 3:**
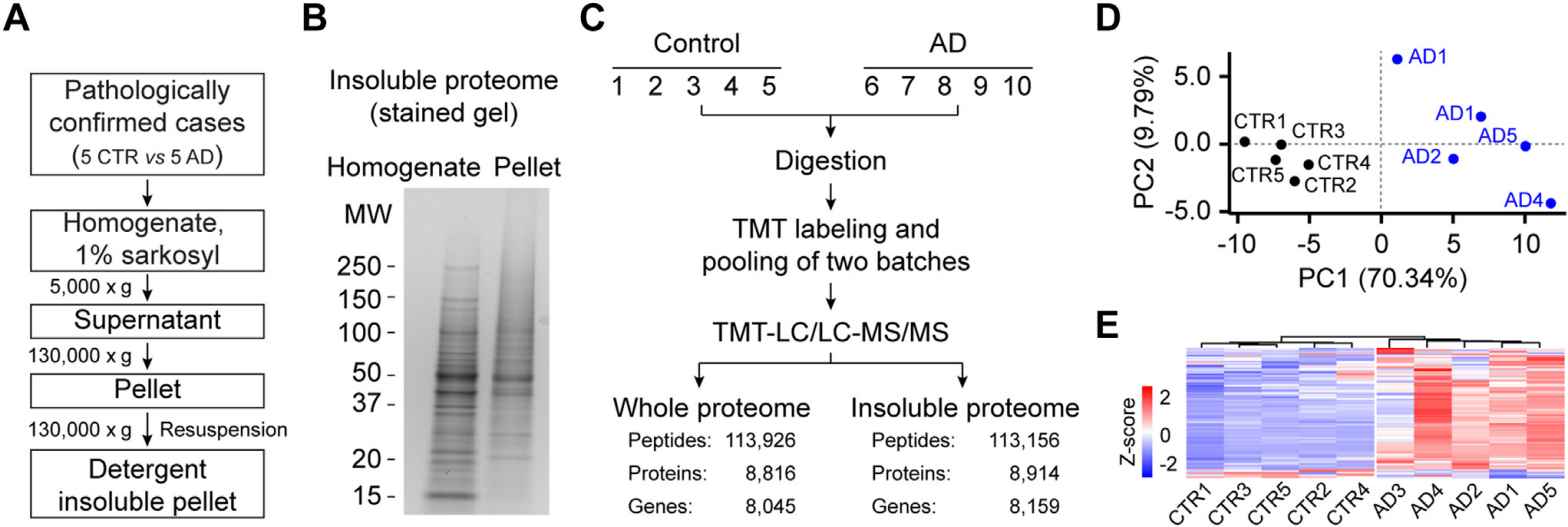
**Detergent-insoluble and whole proteome profiling from human AD brain samples.***A*, sample preparation of detergent-insoluble and whole brain proteome from AD cases and controls. *B*, total homogenate and the detergent-insoluble pellet (∼1 μg per sample) were analyzed by SDS-PAGE with molecular weight (MW) markers (kilodalton) followed by silver staining. *C*, TMT-LC/LC–MS/MS experiments for profiling detergent-insoluble and whole proteomes, in which the overlapped proteins and peptides are 7357 and 82,745, respectively. *D*, principal component analysis (PCA) of the top 1% variable proteins in detergent-insoluble proteome. *E*, heatmap showing the sample clustering with the top 1% most variable proteins in detergent-insoluble proteome. The expression levels were scaled by *z*-score for each protein. AD, Alzheimer's disease.

With this newly developed TMT quantification strategy, we decided to fully characterize the detergent-insoluble proteome of human AD brain. A total of ten human postmortem brain tissue samples were used, including five pathologically confirmed AD cases and five normal controls (supplemental Table S1). To extract the detergent-insoluble proteome, we followed a previously published protocol of sequential centrifugation (21) (Fig. 3*A*). The whole homogenates and the detergent-insoluble fractions were analyzed by SDS gel electrophoresis, exhibiting different patterns of major protein bands (Fig. 3*B*). Both the whole proteome and detergent-insoluble proteome were analyzed by the TMT-LC/LC–MS/MS method, identifying a total of 8816 and 8914 unique proteins below 1% protein FDR, respectively (Fig. 3*C*; supplemental Tables S2 and S3). Principal component analysis and hierarchical clustering of the insoluble proteome indicate the separation of AD and control cases in our proteomics data (Fig. 3, *D* and *E*).

**Fig. 4:**
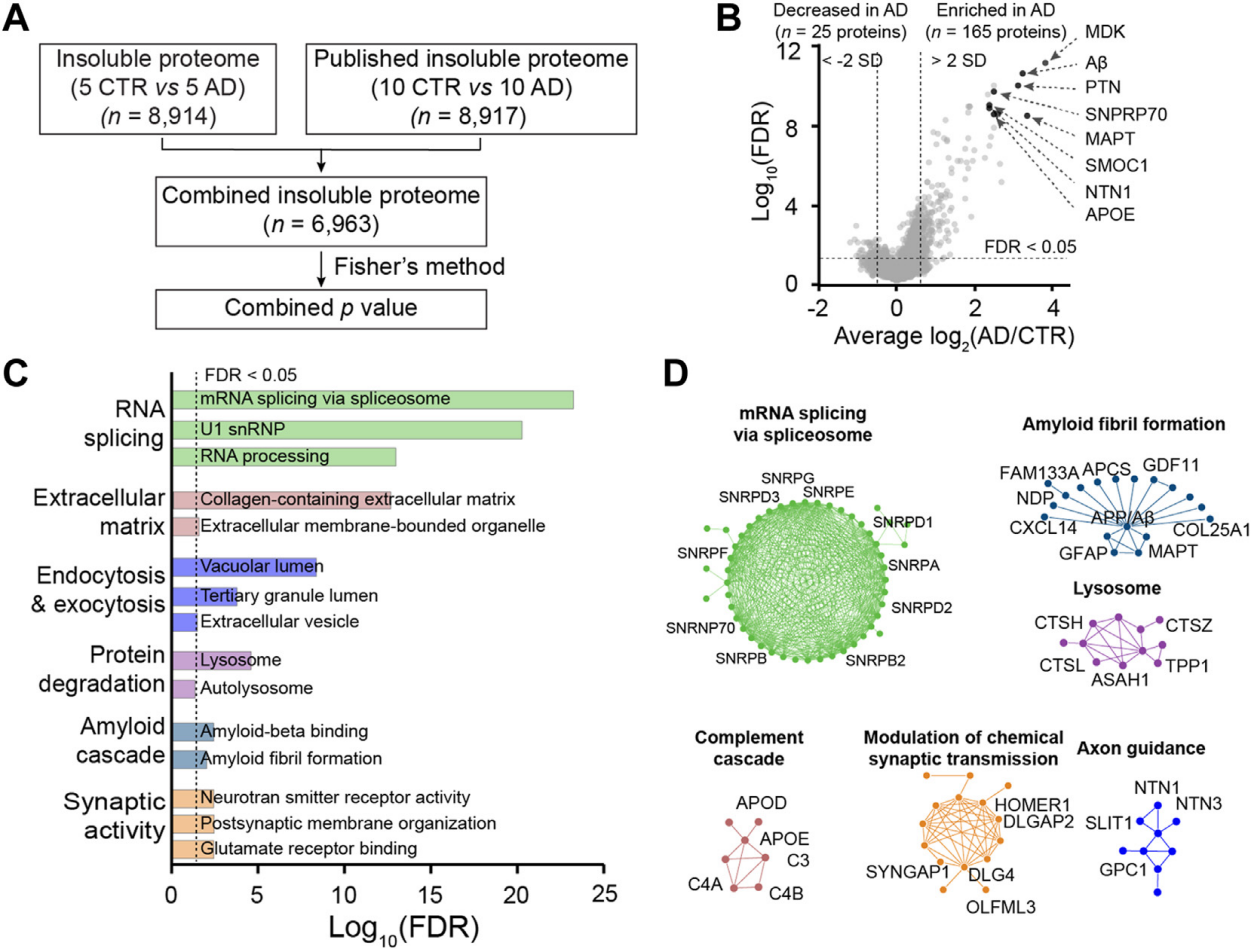
**Differential proteins and pathway enrichment analyses of detergent-insoluble brain proteome.***A*, meta-analysis workflow of the detergent-insoluble brain proteome from two studies. *B*, volcano plot showing DEPs. Each *dot* represents a protein showing log_2_(AD/CTR) and the −log_10_FDR between AD cases and controls. The cutoff was set as FDR <0.05 and log_2_FC >2 average SD that was calculated as the mean of intragroup SDs within five AD cases or five controls. *C*, pathways enriched in DEPs between AD cases and controls (FDR <0.05). Significance of pathway enrichment was performed using two-tailed Fisher's exact test with the Benjamini–Hochberg (BH) multiple testing correction. *D*, representative enriched PPI modules of DEPs. Each *dot* represents a protein, and the interaction is indicated by *connected lines*. AD, Alzheimer's disease; DEP, differentially expressed protein; FC, fold change; FDR, false discovery rate; PPI, protein–protein interaction.

To identify DEPs in the detergent-insoluble proteome between AD and control cases, we performed a meta-analysis with the Fisher’s method by merging current dataset (5 ADs and five controls) and a reported detergent-insoluble proteome (10 ADs and 10 controls) (28) (Fig. 4*A*). Among 6963 proteins detected in both datasets, 190 DEPs were identified between AD and controls (FDR <0.05, |log_2_FC| >0.66 [2 SD], supplemental Table S4), of which 165 proteins were elevated in the AD cases, including Aβ, MAPT, APOE, SNPRP70, MDK, PTN, SMOC1, NTN1, and so on, and 25 proteins were decreased in the AD cases (Fig. 4*B*).

To analyze biological pathways that are significantly enriched for DEPs in detergent-insoluble proteome, we found 90 enriched Gene Ontology terms and Kyoto Encyclopedia of Genes and Genomes pathways, including pathways associated with RNA splicing, extracellular matrix, endocytosis and exocytosis, protein degradation, amyloid cascade, and synaptic activity (Fig. 4*C*; supplemental Table S5). By projecting DEPs to the PPI network, we defined 10 functional modules (Fig. 4*D*; supplemental Table S6). A notable module is mRNA splicing *via* spliceosome, which is composed of the U1 snRNP complex, such as SNRNP70, SNRPA, SNRPB, SNRPD1, SNRPD2, SNRPD3, SNRPE, and SNRPG. U1 snRNP was found to assemble into tangle-like structures in AD brains (21, 26). Consistently, the splicing deficiency was identified in several cohorts of human AD brains by deep RNA sequencing (21, 67).

Considering that aggregated proteins in the AD brains are enriched in the detergent-insoluble proteome, it is reasonable to find that the majority of DEPs (*n* = 165) are increased in AD-insoluble proteome. However, a small number of DEPs (*n* = 25) are decreased in the quantified AD-insoluble proteome, which might represent copurified components during differential extraction. Along this line, some increased DEPs might be detected from copurified components that are not truly aggregated proteins.

### Enriched Proteins in Detergent-Insoluble Proteome Compared with Whole Proteome

**Fig. 5:**
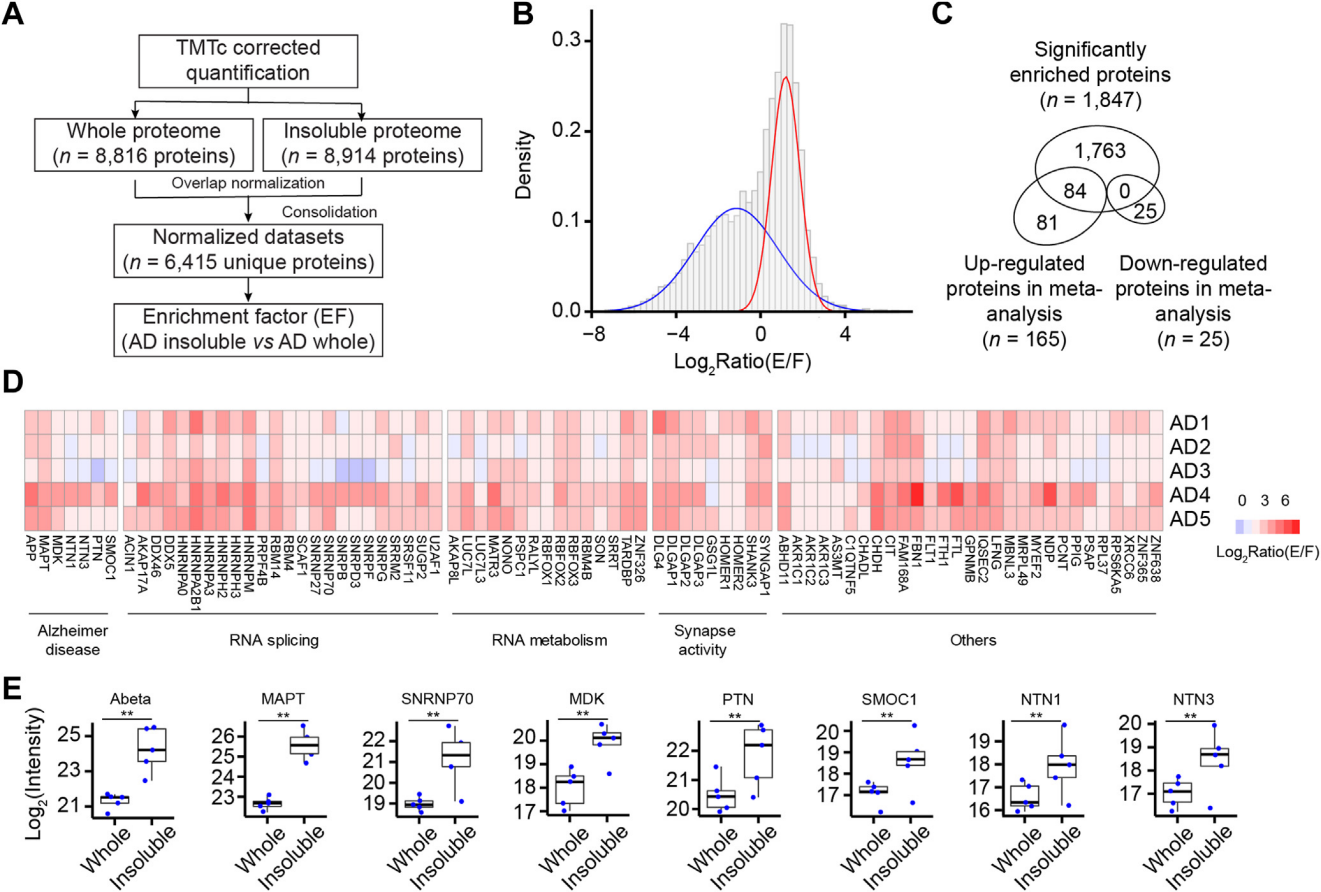
**Enrichment factor analysis of detergent-insoluble brain proteome compared with the whole proteome.***A*, workflow of the enrichment factor analysis. *B*, histogram plot showing the enrichment factor distribution of identified proteins, which fits to a mixed model (two curves of normal distribution). The *x*-axis represents the logarithmic value of enrichment factors between insoluble and whole proteomes. *C*, venn diagram shows the overlap among significantly enriched proteins as well as upregulated and downregulated proteins in AD by the meta-analysis. *D*, heatmap showing enriched and upregulated DEPs (*n* = 84) in individual AD cases. *E*, boxplots showing the levels of some highly enriched proteins in the whole and insoluble proteomes. AD, Alzheimer's disease; DEP, differentially expressed protein.

To distinguish aggregated proteins from copurified components in the protocol of differential extraction, we defined an enrichment factor (EF) for each protein in the detergent-insoluble proteome compared with the whole proteome (Fig. 5*A*, supplemental Table S7). In AD cases, the histogram of the EFs follows a mixed normal distribution, implicating more than one protein population (Fig. 5*B*). After evaluating the experimental variation by intragroup comparisons, we used a cutoff of 2 SD (log_2_EF >1.2) to identify enriched proteins in the insoluble proteome. Strikingly, all decreased 25 DEPs were excluded from the enriched list, confirming that they were simply copurified components (Fig. 5*C*). Of the 165 increased DEPs, 84 proteins stayed in the enriched list, suggesting that they may be aggregated or coaggregated proteins in AD. These 84 enriched proteins showed consistent enrichment in all five individual AD cases, including the pathways of AD, RNA splicing, RNA metabolism, and synaptic activity (Fig. 5*D*). As expected, these enriched proteins include many AD-associated proteins, such as Aβ, MAPT, SNRNP70, MDK, PTN, SMOC1, NTN1, and NTN3 (Fig. 5*E*), all of which showed significant enrichment from the whole proteome to the insoluble proteome.

As protein aggregation is commonly mediated by the interactions of low-complexity regions (LCRs) in amino acid sequences (68), we used the PlaToLoCo metaserver (69) to analyze LCRs in the 84 AD-enriched proteins. Strikingly, 74 (88%) proteins harbor at least one LCR (supplemental Table S8), whereas the proportion of LCR-containing proteins in the Swiss-Prot protein database is estimated to be ∼12% (70). These data strongly support that these AD-enriched proteins in the insoluble proteome favorably possess the biophysical characteristics of LCRs for proaggregation.

In addition, we examined the EF for the proteins in the detergent-insoluble proteome compared with the whole proteome in the control cases (supplemental Fig. S4, supplemental Table S9). Protein EFs from the control cases were remarkably consistent with those from the AD cases (Pearson's *r* = 0.97, supplemental Fig. S5), indicating that the EF of each protein is largely determined by its inherent detergent solubility or insolubility.

### TMT-Labeled Analysis of the Detergent-Insoluble Proteome from AD Cases

**Fig. 6:**
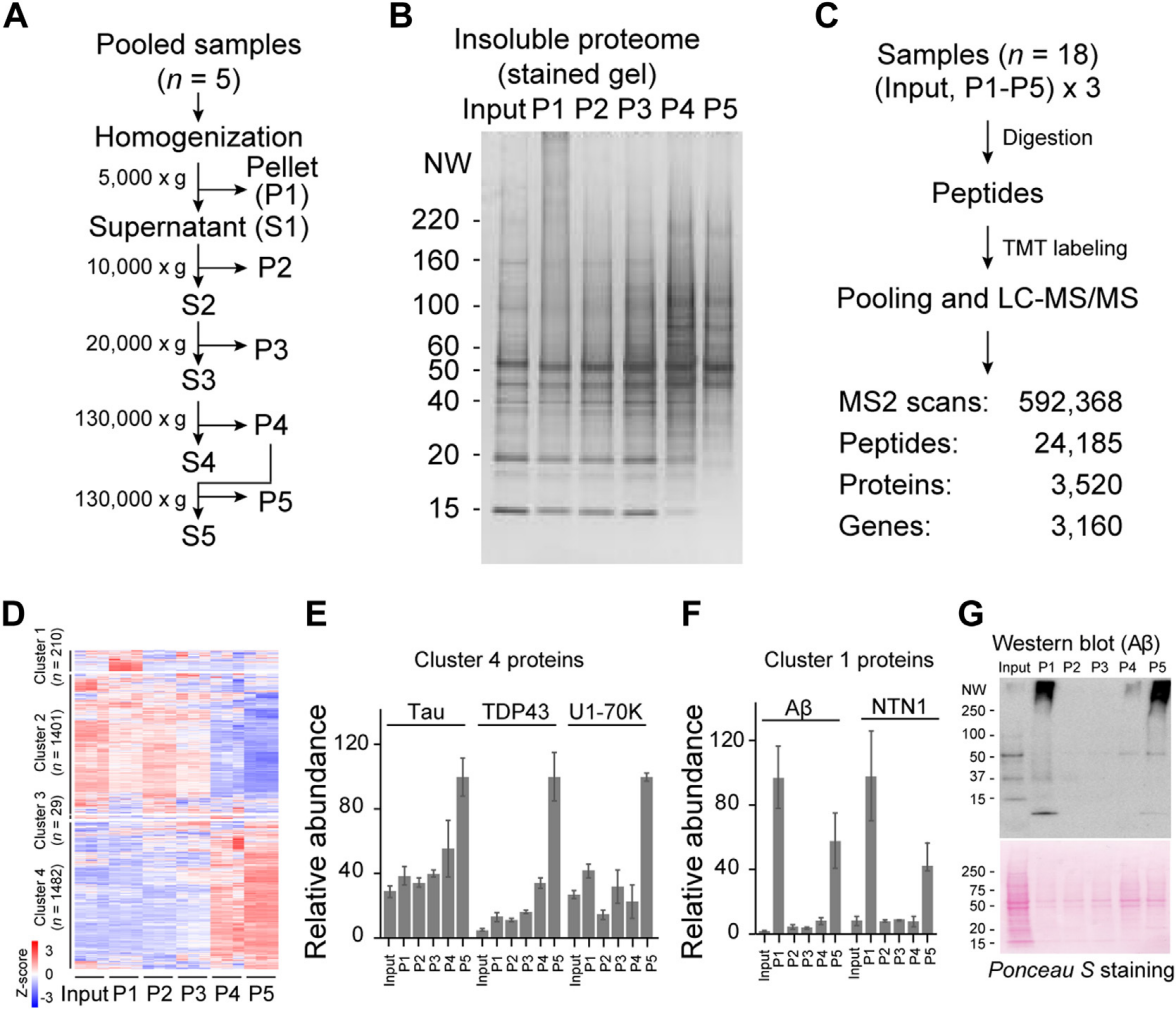
**Validation of enriched proteins using five-step sequential centrifugation.***A*, workflow of sequential extraction protocol used to create detergent-insoluble fractions from a pooled AD brain sample. *B*, silver-stained SDS-PAGE gel of the total homogenate (*input*) and different insoluble fractions obtained from sequential centrifugation steps (∼1 μg per sample). *C*, schematic diagram showing the profiling of AD brain fractions. A total of six biological samples were used, including the total homogenate (input) and five insoluble fractions, each with three replicates. *D*, clustering analysis of the proteins identified with three replicates. *E* and *F*, examples of DEP distribution in the six samples. *G*, Western blot validation of Aβ enrichment in P1 and P5 in different samples (∼5 μg protein per sample except ∼10 μg of the input). Ponceau S staining of the blot indicated the loading level. Aβ, amyloid-beta; AD, Alzheimer's disease; DEP, differentially expressed protein.

To validate DEPs identified in the detergent-insoluble proteome of AD brains, we profiled AD samples during sequential centrifugation with different speeds (Fig. 6*A*). In this experiment, postmortem AD brain samples (frontal cortex) were pooled and homogenized, followed by five centrifugation steps with different speeds, yielding five pellets (P1–P5). The total homogenate (input) and five pellets were analyzed by SDS-PAGE followed by silver staining, showing the change of protein patterns (Fig. 6*B*). To profile all these biochemical fractions, a total of 18 biological samples were analyzed in a TMT-LC/LC–MS/MS experiment, including the input and five detergent-insoluble pellets, each with three replicates (Fig. 6*C*). A total of 3122 unique proteins were identified (<1% protein FDR, supplemental Table S10), and the clustering analysis supported high reproducibility of the replicates (Fig. 6*D*, supplemental Table S11). These proteins can be grouped into four clusters: cluster 1 (*n* = 210, enriched in P1 and also in P5), cluster 2 (*n* = 1401, enriched in the input, P1, P2, and P3), cluster 3 (*n* = 29, enriched in the input and P2), and cluster 4 (*n* = 1482, enriched in P4 and P5).

Among the 84 AD-enriched proteins in the insoluble proteome, 51 proteins were detected in this five-step centrifugation experiment (supplemental Fig. S6*A*), shown only in two clusters: cluster 1 (*n* = 9) and cluster 4 (*n* = 42) (supplemental Fig. S6, *B* and *C*). Interestingly, we observed that the majority of AD-enriched proteins, such as Tau, TDP43, and U1-70K of the U1 snRNP complex, are present in cluster 4 (mostly enriched in P5, Fig. 6*E*). In contrast, some Aβ-associated proteins, including Aβ and NTN1, tend to be present in cluster 1 (enriched in P1 and P5, Fig. 6*F*). The Aβ distribution in the fractions of P1 and P5 was further validated by an orthogonal Western blotting assay (Fig. 6*G*), implicating there are two possible amyloidomes (*i.e.*, Aβ-associated molecules): one large structure such as senile plaques that can be isolated in P1 by 5000*g* centrifugation and another compact structure such as Aβ filaments that can be enriched in P5 by 130,000*g* centrifugation.

## Discussion

To achieve a comprehensive view of the AD-insoluble proteome, we performed a meta-analysis of two insoluble proteome datasets with high coverage (more than 8000 quantified proteins) and define a subset of 84 AD-enriched proteins through AD/control comparison, as well as the analysis of a biochemical EF by detergent insolubility. Importantly, the vast majority of the 84 AD-enriched proteins contain LCRs in protein sequences, consistent with their potential role in protein aggregation.

In this study, we employed the latest TMT method, followed by a novel TMTc-based correction to enhance quantitative accuracy. The TMTc ion quantification eliminates the issue of ratio compression (49, 50, 51), but isobaric TMTc ions can be generated from some adjacent TMT channels (supplemental Fig. S1), and TMTc ions are detected only in a portion of identified spectra and with reduced sensitivity (51). In this study, we observed that approximately 50% of identified PSMs contain a complete set of TMTc ion clusters (*i.e.*, with nine monoisotopic peaks). TMTc ions are primarily generated from doubly charged peptide ions, whereas triply and quadruply charged ions do not produce sufficient TMTc ions. However, since ratio compression does not significantly affect the z value (log_2_FC/SD) of TMT datasets (36, 66), we designed a strategy to correct TMT reporter ion quantification using TMTc ion data and validated this correction strategy with protein samples of known ratios. The strategy may be generally used for computational correction of TMT results.

Our noise correction method enhances protein quantification accuracy in TMT-based proteomics without compromising multiplexity. Unlike the standard TMT, which relies solely on reporter ions, our method considers both reporter and TMTc ions, addressing the ratio compression issue and providing more accurate quantification. The standard TMTc method exclusively uses TMTc ions, limiting quantified sample channels in a single experiment (*e.g.*, nine TMTc ions *versus* 18 TMTpro reporter ions). Our approach employs both TMT reporter and TMTc ions, improving accuracy without sacrificing multiplexity.

We defined a parameter of protein EF to distinguish genuinely enriched components from copurified background proteins during differential centrifugation. The protein EF can be used in any other protein purification experiments to facilitate data interpretation, as profiling all fractions during purification becomes increasingly practical with the improvement of proteomics throughput (44, 45). Of 190 DEPs in AD-insoluble proteome, 84 proteins passed the filter of significant enrichment. In addition to reidentify key disease proteins (*e.g.*, Aβ and Tau), we detected a number of insoluble proteins (*e.g.*, MDK, PTN, NTN1, NTN3, and SMOC1) that were reported to be accumulated in whole proteome profiling (13, 16, 17) and some localized in senile plaques (amyloidome) (11, 13). These proteins may modulate the Aβ precipitation and toxicity during the long-term development of AD pathology (19).

This study also revealed the accumulation of a large number of RNA-binding proteins (*e.g.*, SNRNP70/U1-70K and other U1 snRNP subunits) in AD-insoluble proteome, consistent with previous analysis of the insoluble proteome (21, 23, 28). Apart from core snRNP components, the splicing-associated proteins LUC7L, LUC7L3, and DDX46, each with BAD repeats (a subtype of LCRs) homologous to those of U1-70K, are also enriched among AD-insoluble proteins, suggesting that these BAD-containing proteins shift toward insolubility during AD pathogenesis (23). Moreover, TARDBP/TDP-43 (29), a classic aggregated protein in amyotrophic lateral sclerosis and frontotemporal lobar degeneration, is also identified in the AD-insoluble proteome. Indeed, TDP-43 aggregates were detected in up to ∼50% of AD brains (71), leading to a recently proposed disease type of limbic-predominant age-related TDP-43 encephalopathy (32). These data implicate that these insoluble proteins may interact directly or indirectly, coaggregate, and accelerate disease progress. For example, U1-70K protein is partially colocalized with Tau tangles in AD brains (21). Conversion of soluble Aβ to Aβ oligomers may be cross-seeded by the presence of TDP-43 oligomers, contributing to the formation of Aβ-TDP-43 complexes (72).

In summary, our study has deeply characterized AD detergent-insoluble proteome, which include both well-characterized components of AD pathology and novel potential therapeutic targets. The combination of the TMT-LC/LC–MS/MS technology and TMTc ion–based correction method allows us to address ratio compression in routine MS2-based TMT experiments. Our deep proteomics data and EF analysis generate a core protein list enriched in AD-insoluble proteome, providing a molecular framework for future investigation of molecular interaction, protein coaggregation, cellular toxicity, novel protein pathology, and disease pathogenesis.

## Data availability

Raw MS data have been submitted to the MassIVE repository with accession number MSV000091796. The dataset is also available in the PRIDE repository with number PXD038381.

## Supplemental data

This article contains supplemental data.

## Conflict of interest

The authors declare no competing interests.
