# Supplementary material for: Dissecting Detergent-Insoluble Proteome in Alzheimer's Disease by TMTc-Corrected Quantitative Mass Spectrometry
Source: Mol Cell Proteomics. 2023 Jun 24;22(8):100608. doi: 10.1016/j.mcpro.2023.100608 (PMC10392608; doi:10.1016/j.mcpro.2023.100608)
Supplement: Supplemental Figures S1–S6 and Tables S1–S14 [file mmc1.docx]

**Supplementary Materials for**

**Dissecting detergent-insoluble proteome in Alzheimer's disease by TMTc-corrected quantitative mass spectrometry**

Masihuz Zaman^1,2#^, Yingxue Fu^1,2,3#^, Ping-Chung Chen^1,2^, Huan Sun^1,2^, Shu Yang^1,2^, Zhiping Wu^1,2^, Zhen Wang^1,2^, Suresh Poudel^3^, Geidy E. Serrano^4^, Thomas G. Beach^4^, Ling Li^5^, Xusheng wang^3*^, Junmin Peng^1,2,3*^

^1^Department of Structural Biology, St. Jude Children's Research Hospital, Memphis, TN 38105, USA

^2^Department of Developmental Neurobiology, St. Jude Children's Research Hospital, Memphis, TN 38105, USA

^3^Center for Proteomics and Metabolomics St. Jude Children's Research Hospital, Memphis, TN 38105, USA

^4^Banner Sun Health Research Institute, Sun City, AZ 85351, USA

^5^Department of Biology, University of North Dakota, Grand Forks, ND 58202, USA

*Correspondence: [Xusheng.Wang@STJUDE.ORG](mailto:Xusheng.Wang@STJUDE.ORG), [Junmin.Peng@STJUDE.ORG](mailto:Junmin.Peng@STJUDE.ORG)

*# Contributed equally*





**Supplemental Figure S1. Chemical structures and heavy isotope positions of the 18-plex TMTpro (TMT18) reporter ions and their related TMTc ions.** Some isobaric TMTc ions are generated from multiple TMT channels. The 18-plex TMTpro reagents lead to the formation of 9 TMTc ions. TMTc-ID names (from TMTc-0 to TMTc-8) are defined according to the number of heavy stable isotope labeled atoms.


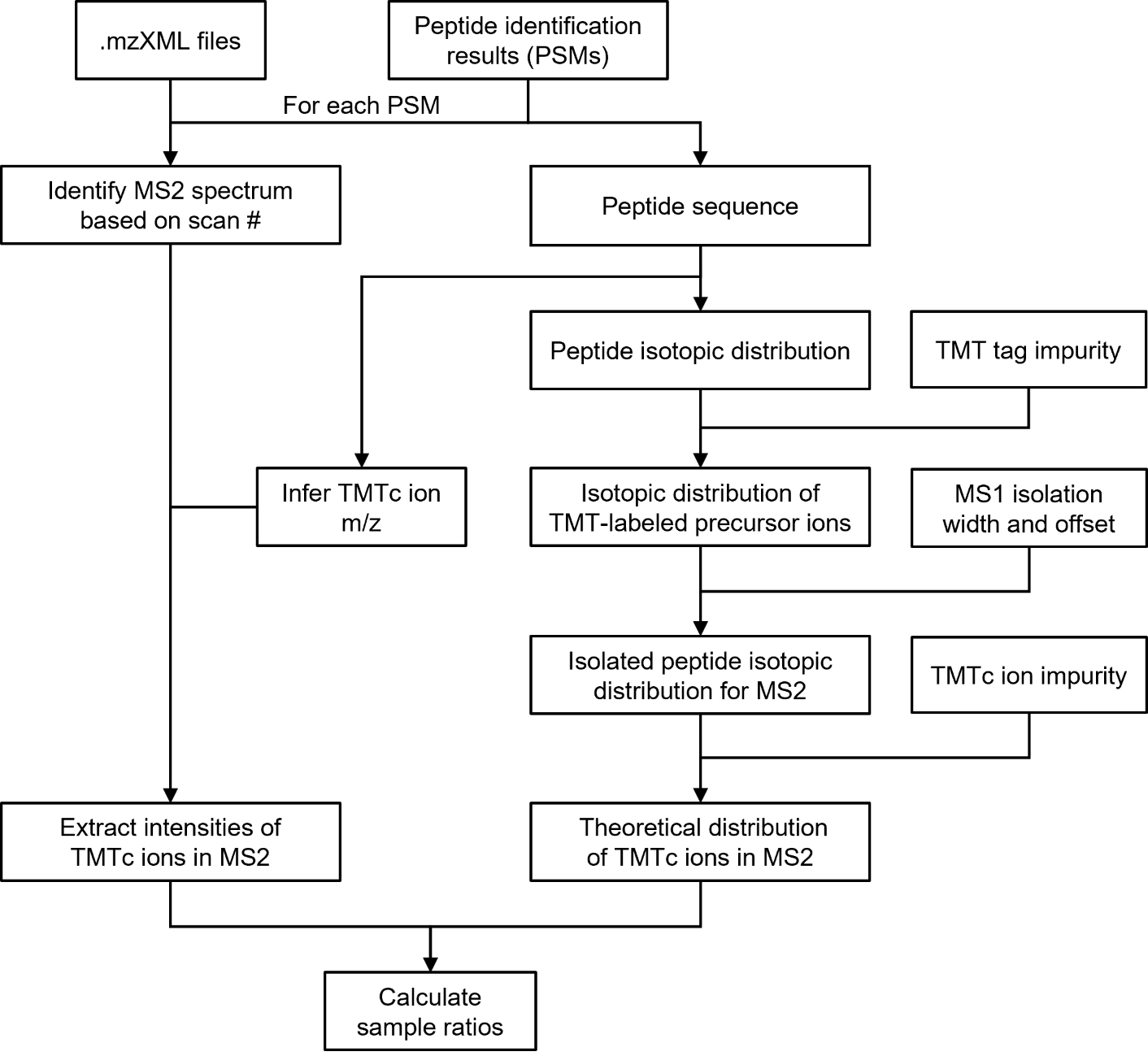


**Supplemental Figure S2. Workflow for quantification based on TMTc ion intensities in the TMT analysis** (see details in **Experimental Procedures**).


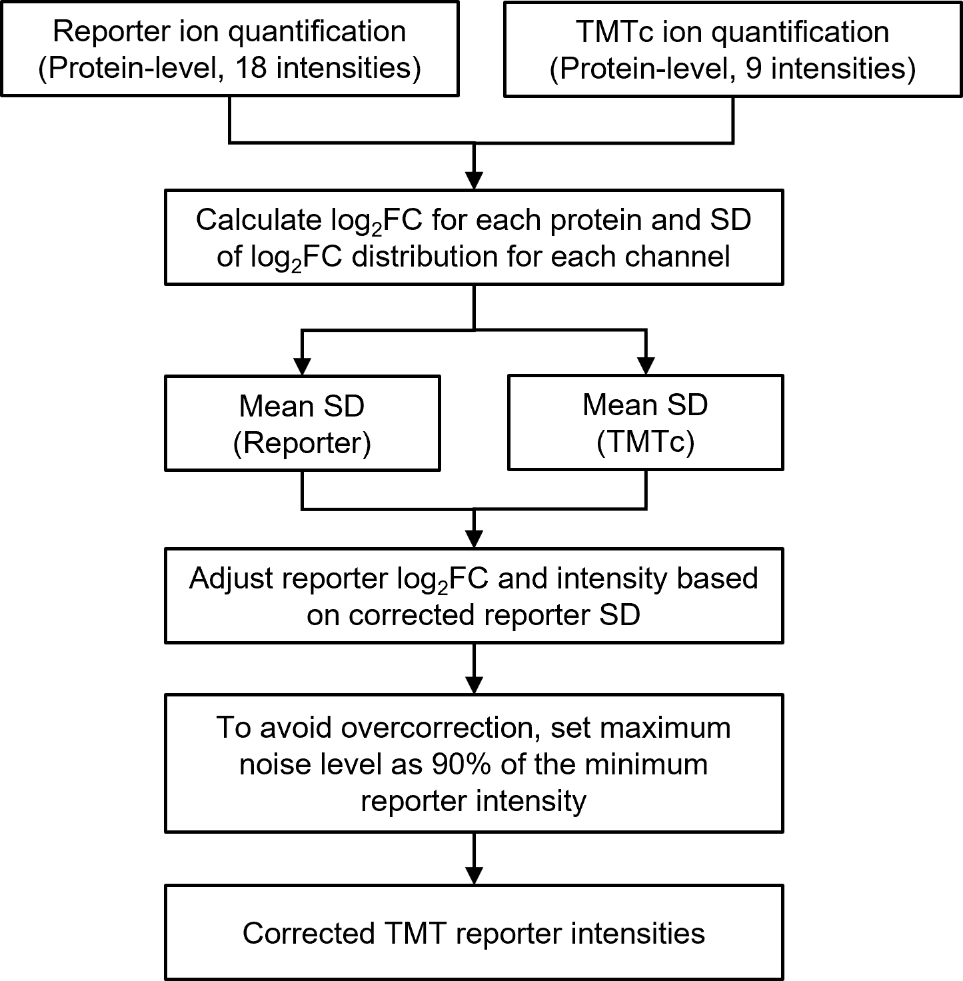


**Supplemental Figure S3. Correction of reporter ion-based quantification by TMTc ion data**.

We first summarized the reporter ion- or TMTc ion-based PSM quantification into protein quantification. The log_2_FC of each protein was calculated between each TMT channel and the average value of all channels. Due to ratio compression in reporter ion quantification, the absolute log_2_FC values are smaller than expected, leading to a smaller mean SD of global log_2_FC distributions. In contrast, TMTc-based quantification is less influenced by interference peptides, resulting in a larger mean SD of global log_2_FC distributions. Assuming the z values remain unchanged between the reporter ion- or TMTc ion-based datasets, we calculated an SD correction factor as the ratio of the two mean SD values. Based on this SD correction factor, we adjusted the reporter SD and corresponding log_2_FC values for all PSMs, including those without TMTc ions. To prevent over-correction, we set the maximum noise level as a portion (e.g., an empirical value of 90%) of the minimum reporter intensity and hypothesized that the noise levels in all 18 channels are the same due to equal loading. Finally, based on the SD-corrected reporter intensity, we calculated a unified noise intensity and subtract this noise from all reporters, generating the final corrected reporter intensities. This noise correction program is publicly accessible on the GitHub repository (<https://github.com/yingxue-fu/tmtc_noise_corrc_MCP>). The program was written in the Python programming language, and detailed instructions on setting up a Conda environment for running the program are provided in the README file. To illustrate the program's functionality, we have included example input files, featuring a raw MS file in mzXML format and its corresponding identification result. Currently, the identification result is text-formatted from our JUMP suite. However, this text format can be easily adjusted to accommodate identification results from other software tools. All source code is publicly available.


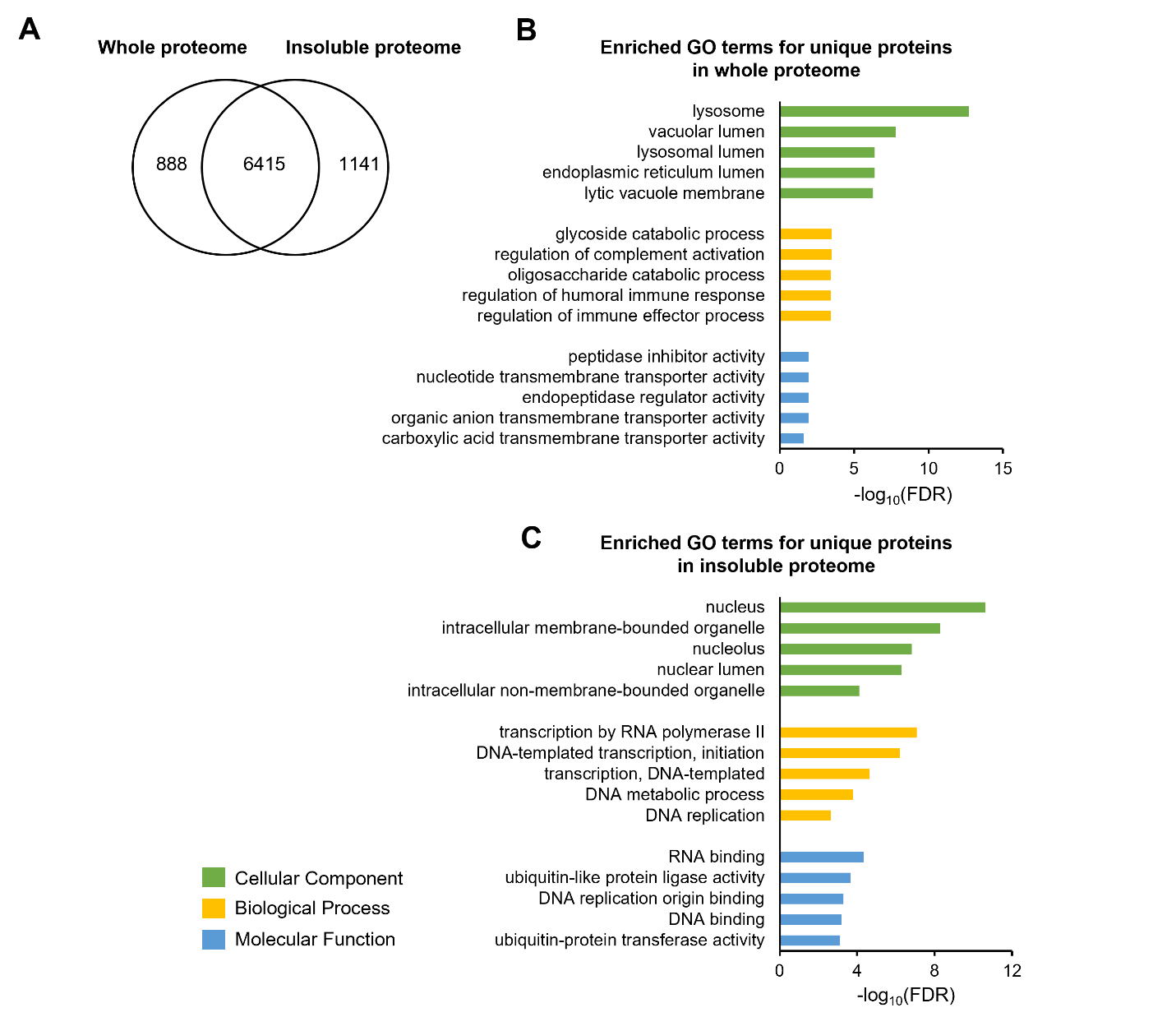


**Supplemental Figure S4. Comparison between detergent-insoluble and whole proteomes from control cases.** (A) Venn diagram showing the number of proteins detected in detergent-insoluble and whole proteomes in the control cases. (B, C) Bar plots showing the enriched GO terms of unique proteins in whole proteome and detergent-insoluble proteome, respectively.


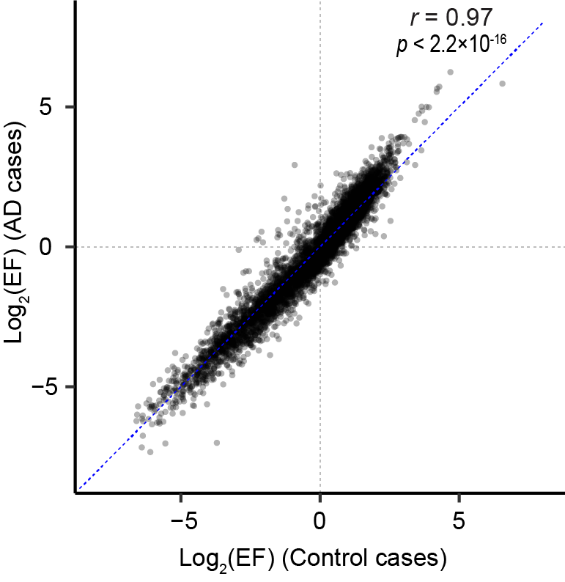


**Supplemental Figure S5. Proteins enriched in detergent-insoluble compared to whole proteome from control cases.** A scatter plot illustrates the comparison of enrichment factors (EF) for proteins found in both AD and control cases. The Pearson correlation coefficient (*r*) and the associated *p* value are shown.


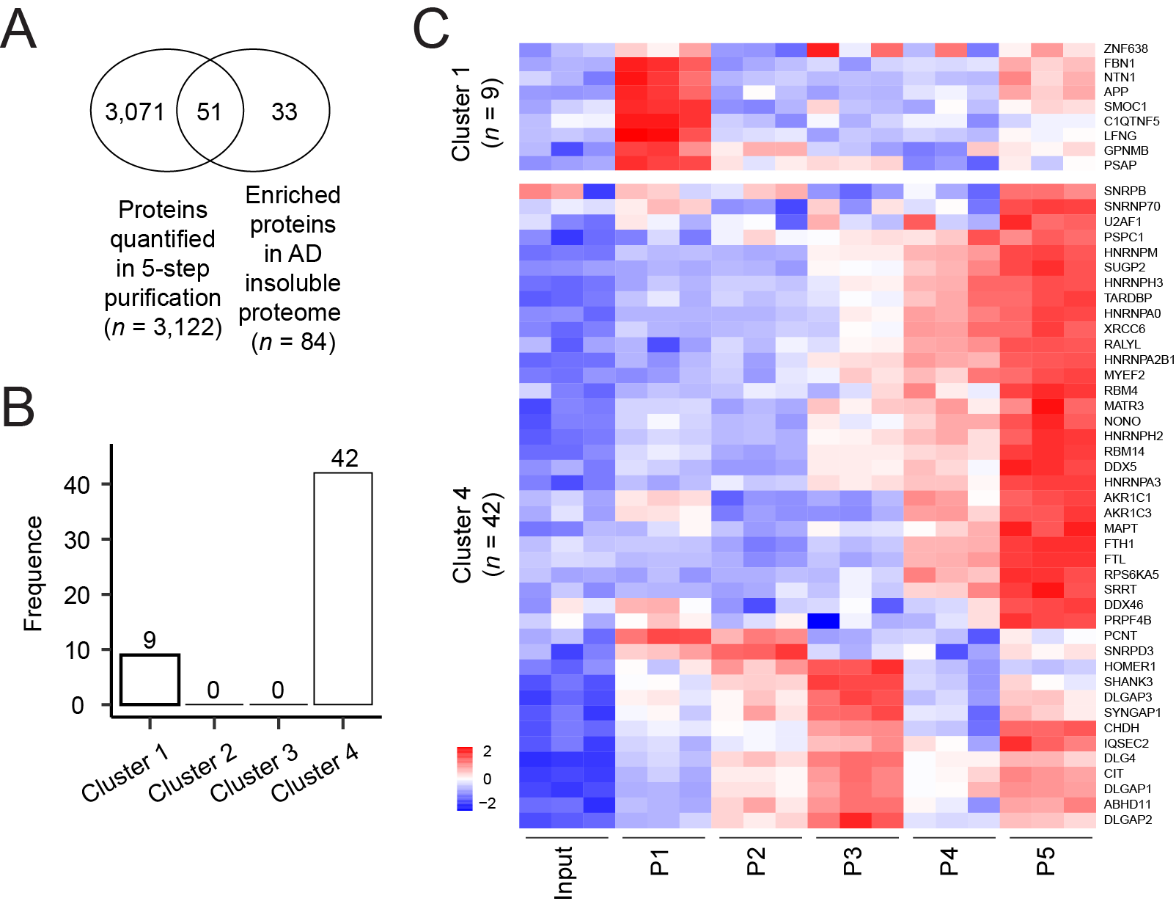


**Supplemental Figure S6. The analysis of the enriched proteins by five-step differential centrifugation.** (A) A total of 51 proteins overlapped between the 84 enriched proteins and the 3,122 proteins identified in the five-step centrifugation. (B) The distribution of the 51 proteins in different clusters. (C) The patterns of the 51 proteins shown in a heatmap.

**Supplementary Tables**

**Table S1.** Human subjects used in this study.

**Table S2.** Whole proteome profiling of AD postmortem human brain tissues by TMT-LC/LC-MS/MS.

**Table S3.** Insoluble proteome profiling of AD postmortem human brain tissues by TMT-LC/LC-MS/MS.

**Table S4.** Meta-analysis of two detergent-insoluble proteomics datasets of AD.

**Table S5.** Functional enrichment analysis of the 165 enriched proteins in detergent-insoluble proteome of AD.

**Table S6.** Protein-protein interaction (PPI) network analysis of the 165 enriched proteins in detergent-insoluble proteome of AD.

**Table S7.** Protein enrichment fold by comparing whole proteome and insoluble proteome.

**Table S8.** Low complexity regions in the 84 enriched proteins in AD insoluble proteome.

**Table S9**. The fold of protein enrichment by comparing whole proteome and insoluble proteome in control cases.

**Table S10.** Proteomic profiling of sequentially centrifuged fractions of human brain tissues.

**Table S11**. Clustering of proteins from five sequential centrifugations.

**Table S12**. Annotated spectra in the pilot experiment to support protein identification by a single unique peptide.

**Table S13**. Annotated spectra in the whole proteome to support protein identification by a single unique peptide.

**Table S14**. Annotated spectra in the detergent-insoluble (aggregated) proteome to support protein identification by a single unique peptide.

**Supplementary Table S8: Low complexity regions in the 84 enriched proteins in AD insoluble proteome.**

LCRs in protein sequences were detected using the SEG method with the PlaToLoCo platform (<http://platoloco.aei.polsl.pl/>). Only 10 proteins do not have LCRs in the protein sequences.

| **Uniprot ID** | **Gene Name** | **Log_2_EF** | **Location of LCRs** |
| --- | --- | --- | --- |
| P35555 | FBN1 | 3.70 | 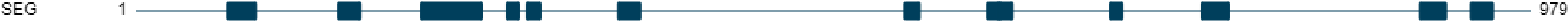 |
| Q02040 | AKAP17A | 2.99 | 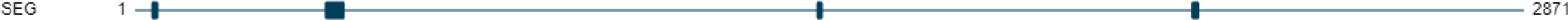 |
| Q96PK6 | RBM14 | 2.80 | 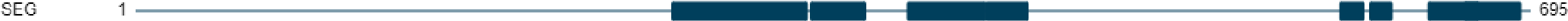 |
| P55795 | HNRNPH2 | 3.27 | 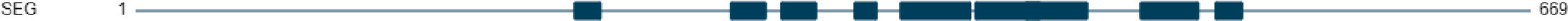 |
| Q00604 | NDP | 2.88 | 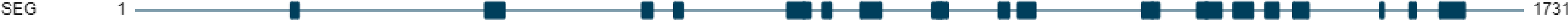 |
| P78352 | DLG4 | 3.36 | 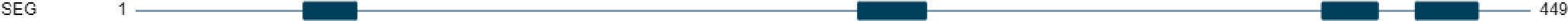 |
| Q5JU85 | IQSEC2 | 3.43 | 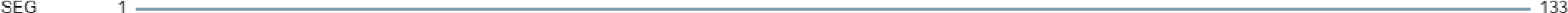 |
| Q8NES3 | LFNG | 2.95 | 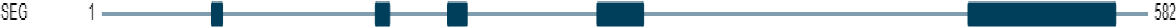 |
| Q92567 | FAM168A | 3.51 | 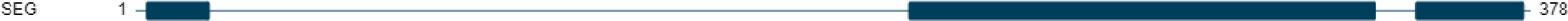 |
| P17844 | DDX5 | 3.08 | 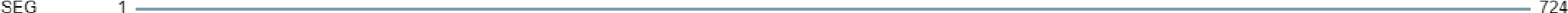 |
| O14578 | CIT | 3.35 | 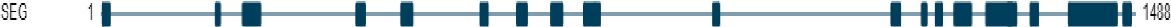 |
| P52272 | HNRNPM | 3.67 | 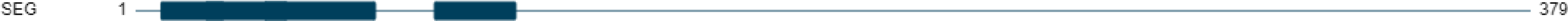 |
| P43243 | MATR3 | 3.06 | 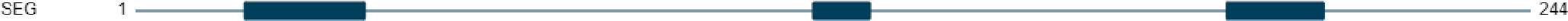 |
| Q8NFV4 | ABHD11 | 2.76 | 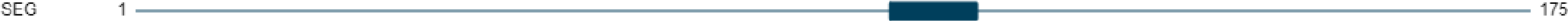 |
| P22626 | HNRNPA21 | 3.93 | 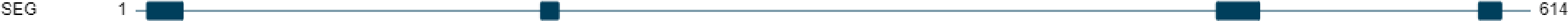 |
| Q13148 | TARDBP | 3.09 | 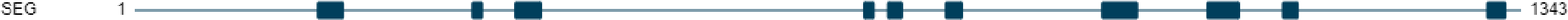 |
| P10636 | MAPT | 2.92 | 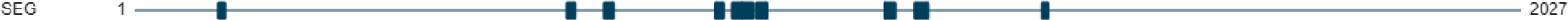 |
| O14490 | DLGAP1 | 3.06 | 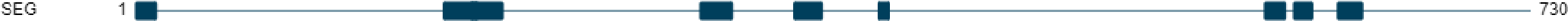 |
| P05067 | APP | 2.92 | 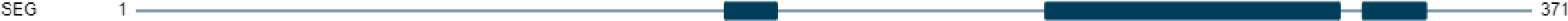 |
| Q8NE62 | CHDH | 3.38 | 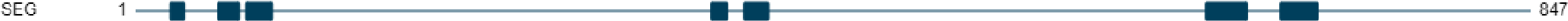 |
| O95886 | DLGAP3 | 2.56 | 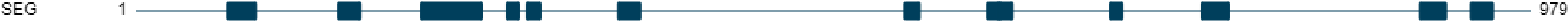 |
| Q9BYB0 | SHANK3 | 2.74 | 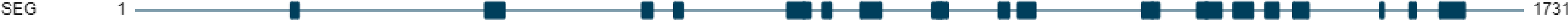 |
| P02794 | FTH1 | 2.15 | 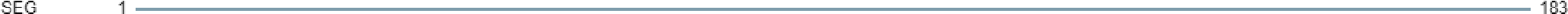 |
| Q5BKZ1 | ZNF326 | 2.72 | 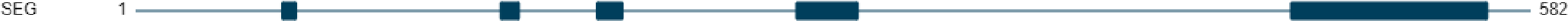 |
| O95613 | PCNT | 2.10 | 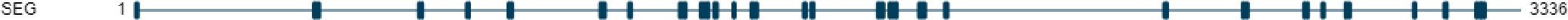 |
| P51991 | HNRNPA3 | 2.73 | 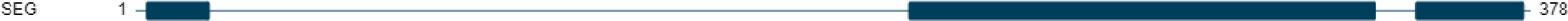 |
| Q15233 | NONO | 2.42 | 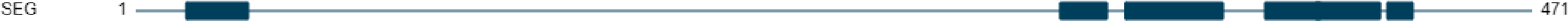 |
| P02792 | FTL | 2.63 | 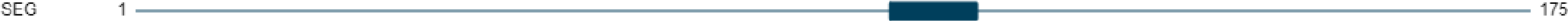 |
| Q13151 | HNRNPA0 | 2.44 | 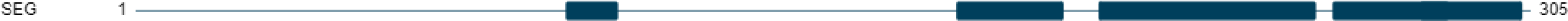 |
| Q14956 | GPNMB | 2.19 | 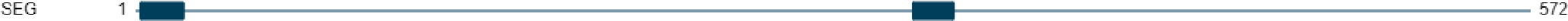 |
| Q9P1A6 | DLGAP2 | 2.38 | 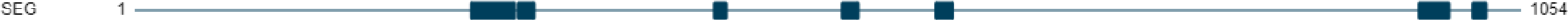 |
| Q96PV0 | SYNGAP1 | 2.77 | 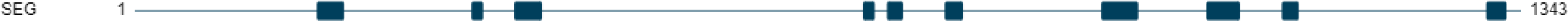 |
| P08621 | SNRNP70 | 2.19 | 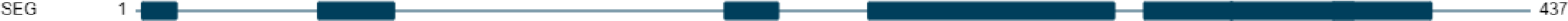 |
| Q9NQ29 | LUC7L | 2.60 | 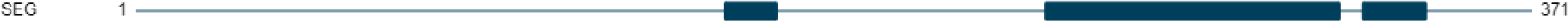 |
| Q8IX01 | SUGP2 | 2.64 | 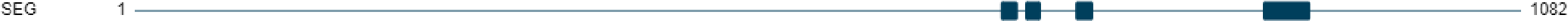 |
| Q9P2K5 | MYEF2 | 2.23 | 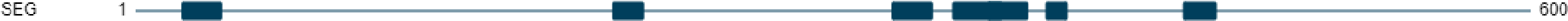 |
| O43251 | RBFOX2 | 2.68 | 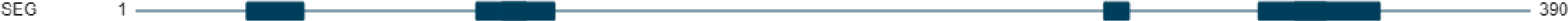 |
| P31942 | HNRNPH3 | 2.44 | 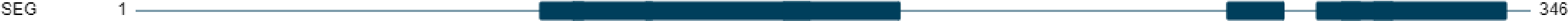 |
| Q9NUK0 | MBNL3 | 2.73 | 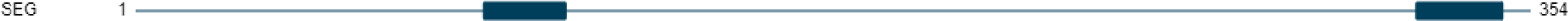 |
| A6NFN3 | RBFOX3 | 2.29 | 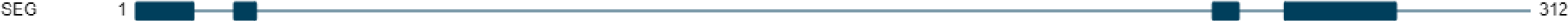 |
| Q01081 | U2AF1 | 1.86 | 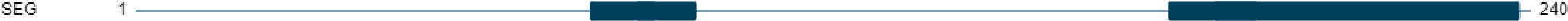 |
| Q14966 | ZNF638 | 1.87 | 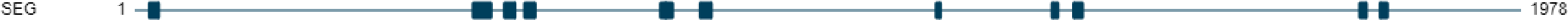 |
| Q9BQ04 | RBM4B | 1.76 | 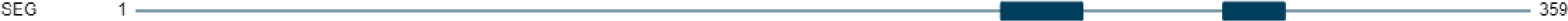 |
| P12956 | XRCC6 | 1.95 | 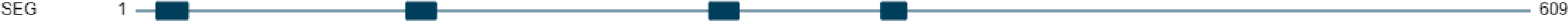 |
| P62308 | SNRPG | 1.80 | 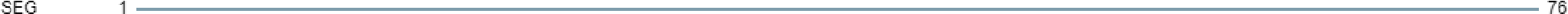 |
| Q86SE5 | RALYL | 1.93 | 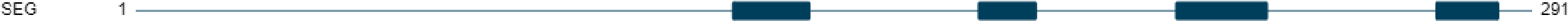 |
| Q9BXP5 | SRRT | 1.82 | 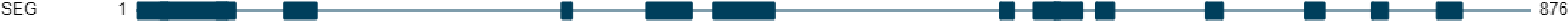 |
| Q9UQ35 | SRRM2 | 1.94 | 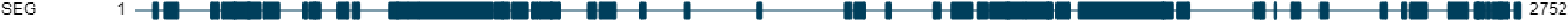 |
| O75582 | RPS6KA5 | 1.91 | 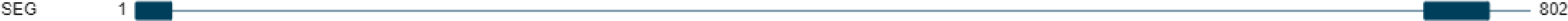 |
| P07602 | PSAP | 1.77 | 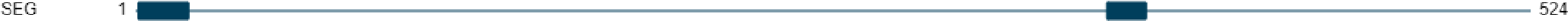 |
| P21741 | MDK | 1.91 | 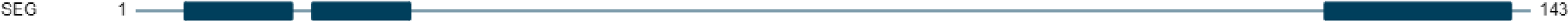 |
| Q04828 | AKR1C1 | 1.68 | 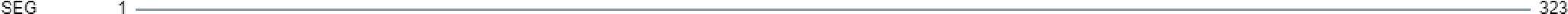 |
| Q13405 | MRPL49 | 2.07 | 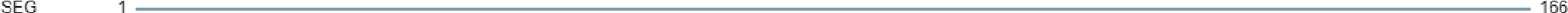 |
| Q7L014 | DDX46 | 1.97 | 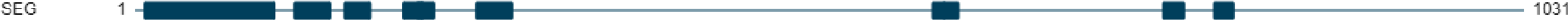 |
| Q9BWF3 | RBM4 | 1.85 | 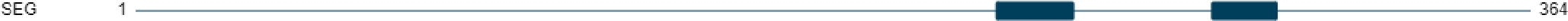 |
| Q9HBK9 | AS3MT | 1.95 | 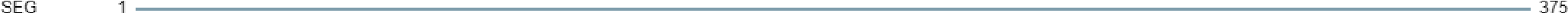 |
| Q13427\| | PPIG | 2.06 | 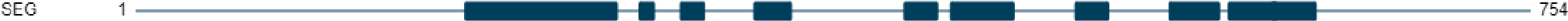 |
| Q8WXF1 | PSPC1 | 1.89 | 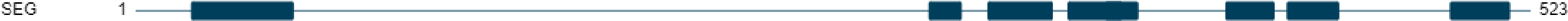 |
| Q86YM7 | HOMER1 | 1.66 | 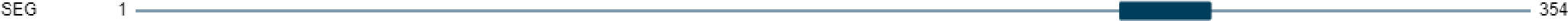 |
| Q70YC5 | ZNF365 | 2.05 | 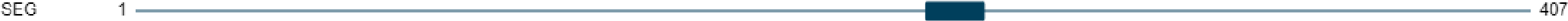 |
| P14678 | SNRPB | 1.23 | 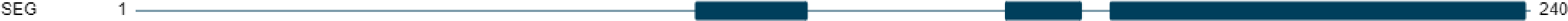 |
| P61927 | RPL37 | 1.25 | 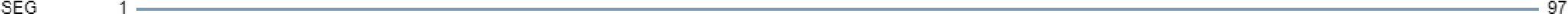 |
| Q9H4F8 | SMOC1 | 1.50 | 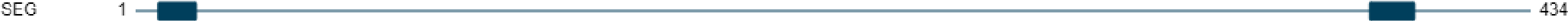 |
| O95631 | NTN1 | 1.37 | 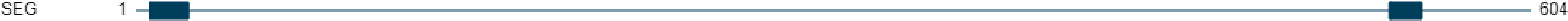 |
| P62306 | SNRPF | 1.48 | 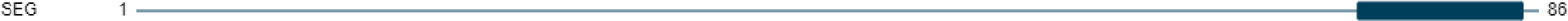 |
| Q8WVK2 | SNRNP27 | 1.57 | 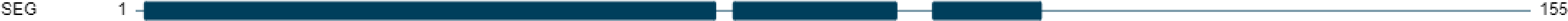 |
| Q9ULX6 | AKAP8L | 1.65 | 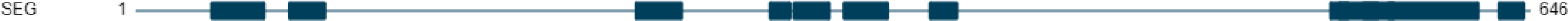 |
| P42330 | AKR1C3 | 1.59 | 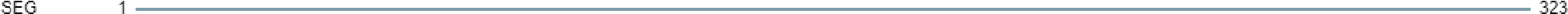 |
| P52895 | AKR1C2 | 1.43 | 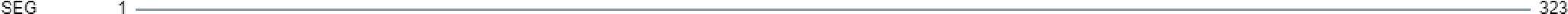 |
| Q13523 | PRPF4B | 1.65 | 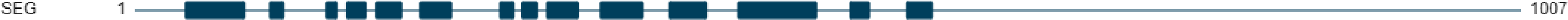 |
| Q9H7N4 | SCAF1 | 1.41 | 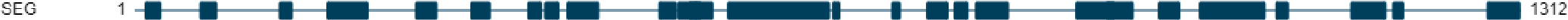 |
| Q9NSB8 | HOMER2 | 1.63 | 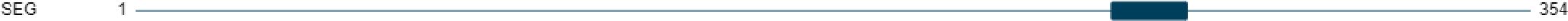 |
| O95232 | LUC7L3 | 1.29 | 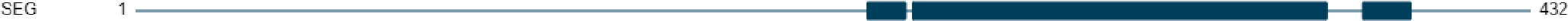 |
| P21246 | PTN | 1.35 | 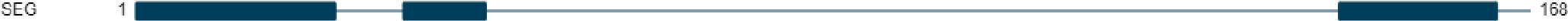 |
| Q05519 | SRSF11 | 1.45 | 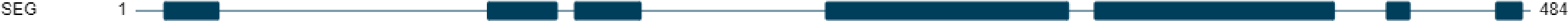 |
| Q9BXJ0 | C1QTNF5 | 1.45 | 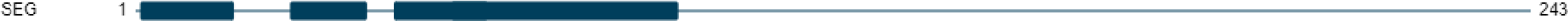 |
| Q9NWB | RBFOX1 | 1.56 | 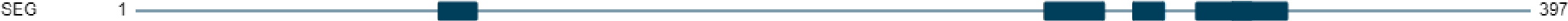 |
| Q6NUI6 | CHADL | 1.33 | 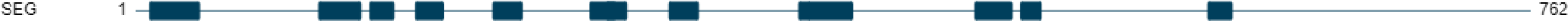 |
| P17948 | FLT1 | 1.59 | 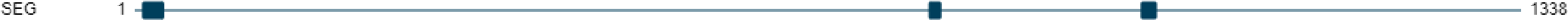 |
| P62318 | SNRPD3 | 1.37 | 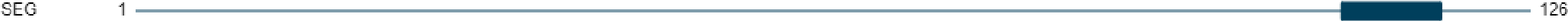 |
| P18583 | SON | 1.48 | 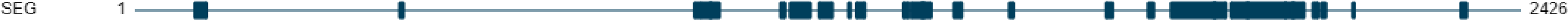 |
| Q9UKV3 | ACIN1 | 1.43 | 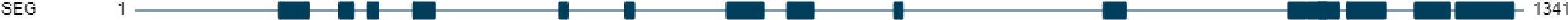 |
| O00634 | NTN3 | 1.39 | 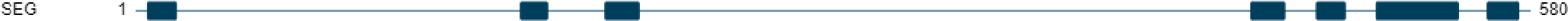 |
| Q6UXU4 | GSG1L | 1.40 | 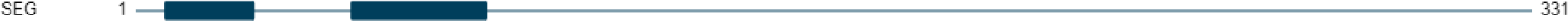 |
